# An 11-Gene Signature Risk-Prediction Model Based on Prognosis-Related miRNAs and Their Target Genes in Lung Adenocarcinoma

**DOI:** 10.3389/fonc.2021.726742

**Published:** 2021-11-05

**Authors:** Ning Zhou, Min Zhou, Ning Ding, Qinglin Li, Guangming Ren

**Affiliations:** Department of Respiratory Medicine, The Affiliated Xuzhou City Hospital of Xuzhou Medical University, Xuzhou, China

**Keywords:** lung adenocarcinoma, miRNA, prognosis, LASSO, risk score

## Abstract

Aberrant expression of microRNAs may affect tumorigenesis and progression by regulating their target genes. This study aimed to construct a risk model for predicting the prognosis of patients with lung adenocarcinoma (LUAD) based on differentially expressed microRNA-regulated target genes. The miRNA sequencing data, RNA sequencing data, and patients’ LUAD clinical data were downloaded from the The Cancer Genome Atlas (TCGA) database. Differentially expressed miRNAs and genes were screened out by combining differential analysis with LASSO regression analysis to further screen out miRNAs associated with patients’ prognosis, and target gene prediction was performed for these miRNAs using a target gene database. Overlapping gene screening was performed for target genes and differentially expressed genes. LASSO regression analysis and survival analysis were then used to identify key genes. Risk score equations for prognostic models were established using multifactorial COX regression analysis to construct survival prognostic models, and the accuracy of the models was evaluated using subject working characteristic curves. The groups were divided into high- and low-risk groups according to the median risk score, and the correlation with the clinicopathological characteristics of the patients was observed. A total of 123 up-regulated miRNAs and 22 down-regulated miRNAs were obtained in this study. Five prognosis-related miRNAs were screened using LASSO regression analysis and Kaplan-Meier method validation, and their target genes were screened with the overlap of differentially expressed genes before multifactorial COX analysis finally resulted in an 11-gene risk model for predicting patient prognosis. The area under the ROC curve proved that the model has high accuracy. The 11-gene risk-prediction model constructed in this study may be an effective predictor of prognosis.

## Introduction

Despite advances in lung cancer treatment, in 2020, lung cancer remained the deadliest type of cancer worldwide according to the World Health Organization (WHO) (1). The 5-year survival rate for lung cancer patients is only 19 percent (2). Part of the reason for the short survival rate in lung cancer mainly contributes to the lung cancer-associated pulmonary hypertension caused by blockage of pulmonary blood vessels due to cancer cell proliferation, which eventually leads to death (3, 4). Lung adenocarcinoma (LUAD) belongs to a subtype of non-small cell lung cancer (NSCLC), and NSCLC accounts for another 85 percent of all lung cancers (5). Most patients with LUAD have an advanced or metastatic disease at the time of diagnosis (6); therefore, early diagnosis is crucial, prolonging patient survival and significantly improving survival rates. LUAD is highly heterogeneous at multiple clinical, behavioural, cellular, and molecular levels (7, 8). The cellular and molecular mechanisms regarding the biological behaviour of tumours remain largely unknown.

Aberrant expression of miRNAs in several cancers, including lung cancer, is associated with tumorigenesis and progression (9, 10). With the application of gene sequencing in tumours, miRNAs can be considered as new biomarkers in patient prognosis prediction and drug resistance (11–13). In addition, integration of multiple miRNAs may be more efficient than a single miRNA for prognosis prediction (14, 15). A large number of studies have identified a series of miRNA signatures that can serve as potential biomarkers for LUAD patient prognosis prediction (15, 16). It is well known that miRNAs have important roles in the regulation of gene expression, either through a single miRNA that regulates the expression of multiple genes or through the combination of several miRNAs that finely regulate the expression of a gene, which in turn regulates various physiological processes, cellular functions, and signalling pathways (17, 18). Studies have been conducted to identify key miRNAs and hub genes in LUAD by bioinformatics and functional analysis (19), Li X et al. identified prognostic biomarkers in lung adenocarcinoma based on aberrant lncRNA–miRNA–mRNA networks and Cox regression models. Gu et al. (20),constructed a DElncRNA-DEmiRNA-DEmRNA ceRNA network for deeper understanding the underlying molecular mechanism of lung adenocarcinoma and for evaluating prognosis. But not much research has been reported on the construction of patient prognostic predictive risk models based on differentially expressed miRNA-target gene-differentially expressed gene networks in LUAD.

In this study, we first analysed the expression profiles of miRNAs in the TCGA database for LUAD to obtain differentially expressed miRNAs. Those miRNAs associated with patient prognosis were then screened by LASSO regression analysis and KM validation, and their target genes were combined with differentially expressed genes in LUAD to construct a prognostic risk-prediction model and verify the validity of the risk model. The patients were then divided into two groups according to risk scores – high risk and low risk – and the correlation between different risk groups and clinicopathological characteristics of LUAD patients was observed to assess the prognostic significance of this risk-prediction model in LUAD.

## Methods

### Data Collection

The miRNA-seq, RNA-seq, and clinical information of lung cancer (LUAD) were obtained by downloading from the TCGA database. If there were multiple probes to detect the same miRNA expression during the analysis, the average of the miRNA expression was taken as an expression value of the miRNA. For the analysis of patient clinical information, clinical information of patients with unknown survival time and survival time of 0 was deleted. The independent validation cohort GSE50081 (21) were obtained from the Gene Expression Omnibus (https://www.ncbi.nlm.nih.gov/geo) by the GEO query R package.

### Differential Analysis

Differentially expressed miRNAs in LUAD were screened using the edgeR package in R software with |logFC|≧1, adjust P value < 0.05.Due to the large amount of differentially expressed genes, we changed the cut-off values with |logFC|≧2, adjust P value < 0.05 for the screen of differentially expressed genes. Volcano plots of differentially expressed miRNAs and genes were plotted using ggplot2.

### Target Gene Prediction

The miRTarBase (http://miRTarBase.cuhk.edu.cn/), TargetScan (http://www.targetscan.org/vert_72/) and RNA22 (https://cm.jefferson.edu/rna22/) target gene prediction databases were used for target gene prediction of miRNAs.

### Kaplan-Meier Survival Analysis

Survival analysis was performed using Survival in the R package. p-values and hazard ratios (HR) with 95% confidence intervals (CI) in Kaplan-Meier curves were derived by log rank test and univariate Cox proportional hazards regression.

### LASSO/Cox Regression Analysis

The LASSO regression algorithm was used for feature selection, and a 10-fold cross-validation was used to determine the parameters and obtain a suitable model. The genes obtained from LASSO regression were then subjected to multifactorial Cox regression analysis, and the multifactorial regression coefficients were calculated for each gene to construct the risk score equation.

### Gene Ontology Enrichment Analyses

GO (is a recognized bioinformatics tool for annotating genes and the analysis of the biological process of target genes.7 To explore the function of 11 genes, biological analysis was performed using DAVID online database. P<0.05 was considered statistically significant.

### Establishment and Analysis of Risk Prognostic Model

Based on the results of the above multi-factor Cox regression analysis, the risk score equation based on gene expression was constructed. Based on the median value of the risk score values, LUAD patients were divided into high-risk score groups and low-risk score groups. Column plots of the model predicting prognosis were drawn using R software, and ROC curves and calibration curves of the model were plotted to evaluate the sensitivity and specificity of the model.

### Statistical Analysis

R3.6.3 was used for all statistical analyses. Values of p<0.05 were defined as statistically significant. In the survival analysis, the survival outcome was defined as overall survival based on clinical record. Univariate and multivariate cox regression analyses were used to assess the influences of the genes on patients ‘survival.

## Results

### Differential Expression miRNA Screening

**Figure 1:**
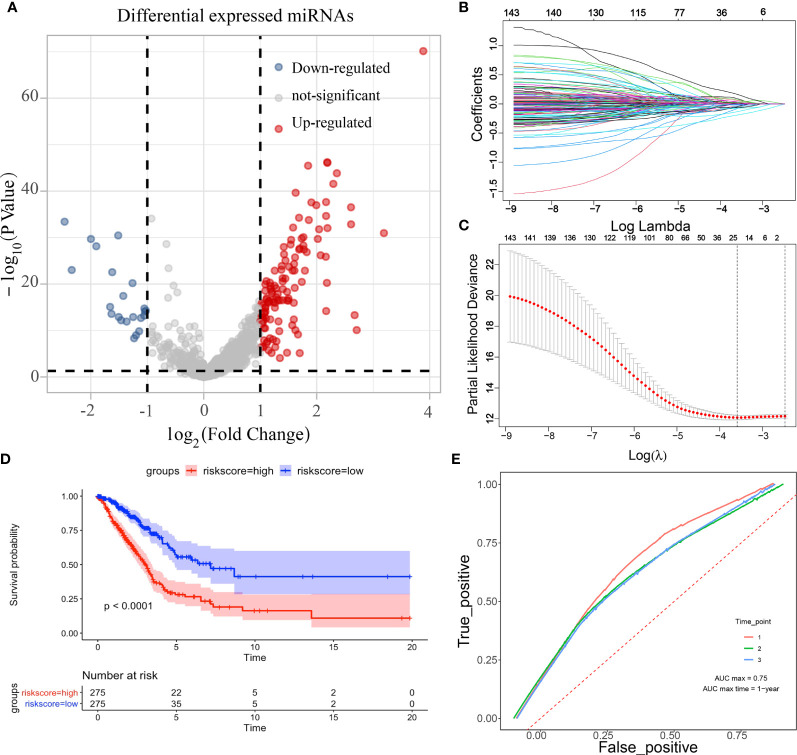
Differential expression miRNA screening. (**A**: volcano plot showing differentially expressed miRNAs; **B**: coefficients of selected features shown by lambda parameters; **C**: partial likelihood deviation versus log(λ); **D**: survival curves of high-and low-risk samples under LASSO regression; **E**; ROC curves under LASSO regression).

The edgeR package was applied to analyse differential miRNA expression in LUAD, and the results were demonstrated by the volcano plot (**Figure 1A**). A total of 123 up-regulated miRNAs and 22 down-regulated miRNAs were obtained. Further screening of these miRNAs using LASSO regression (**Figure 1B**) and modelling using cross-validation (**Figure 1C**) was conducted. A total of 23 miRNAs associated with prognosis (hsa-miR-450a-5p, hsa-miR-548v, hsa-miR-490-3p, hsa-miR-142-3p, hsa-miR-20a-5p, hsa-miR-323a-3p, hsa-miR-301b-5p, hsa-miR-940, hsa-miR-550a-5p, hsa-miR-106a-5p, hsa-miR-3653-5p, hsa-let-7c-5p, hsa-miR-31-5p, hsa-miR-137, hsa-miR-192-5p, hsa-miR-642a-5p, hsa-miR-148a-3p, hsa-miR-5698, hsa-miR-196b-5p, hsa-miR-3607-3p, hsa-miR-9-3, hsa-let-7g-3p, hsa-miR-31-3p) were obtained by constituting a multivariate linear model, which was divided into high and low risk according to the prognostic index of each sample, and KM curves showed that high- and low-risk patients survived with significant differences (**Figure 1D**). To verify the accuracy of this prognostic model, it was further corroborated by the ROC curves, and the results showed that the prognostic model performed well (**Figure 1E**).

### Kaplan-Meier Method to Validate 23 miRNAs

**Figure 2:**
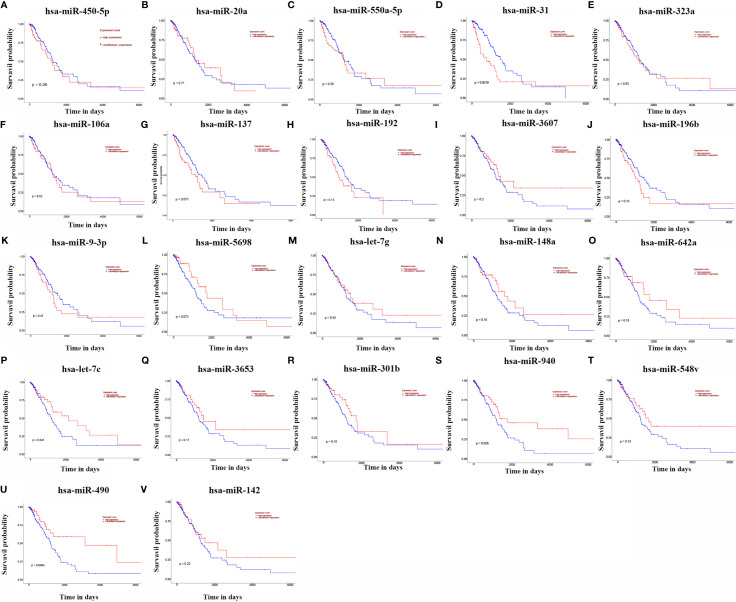
KM curves of 23 miRNAs. (**A**: hsa-miR-450a-5p; **B**: hsa-miR-20a; **C**: hsa-miR-550a-5p; **D**: hsa-miR-31; **E**: hsa-miR-323a; **F**: hsa-miR-106a; **G**: hsa-miR-137; **H**: hsa-miR-192; **I**: hsa- miR-3607; **J**: hsa-miR-196b; **K**: hsa-miR-9-3p; **L**: hsa-miR-5698; **M**: hsa-let-7g; **N**: hsa-miR-148a; **O**: hsa-miR-642a; **P**: hsa-let-7c; **Q**: hsa-miR-3653; **R**: hsa -miR-301b; **S**: hsa-miR-940; **T**: hsa-miR-548v; **U**: hsa-miR-490; **V**: hsa-miR-142).

The relationship between the expression of 23 miRNAs and the survival prognosis of LUAD patients was analysed using the UALCAN database (**Figures 2A–V**). The results showed that the expression of hsa-miR-490-3p, hsa-miR-940, hsa-miR-31-3p, hsa-miR-31-5p and hsa-let-7c-5p were significantly associated with the survival prognosis of LUAD patients, and high expression of these miRNAs was associated with poor prognosis of LUAD patients.

### Screening of miRNAs Target Genes and Differentially Expressed Genes

**Figure 3:**
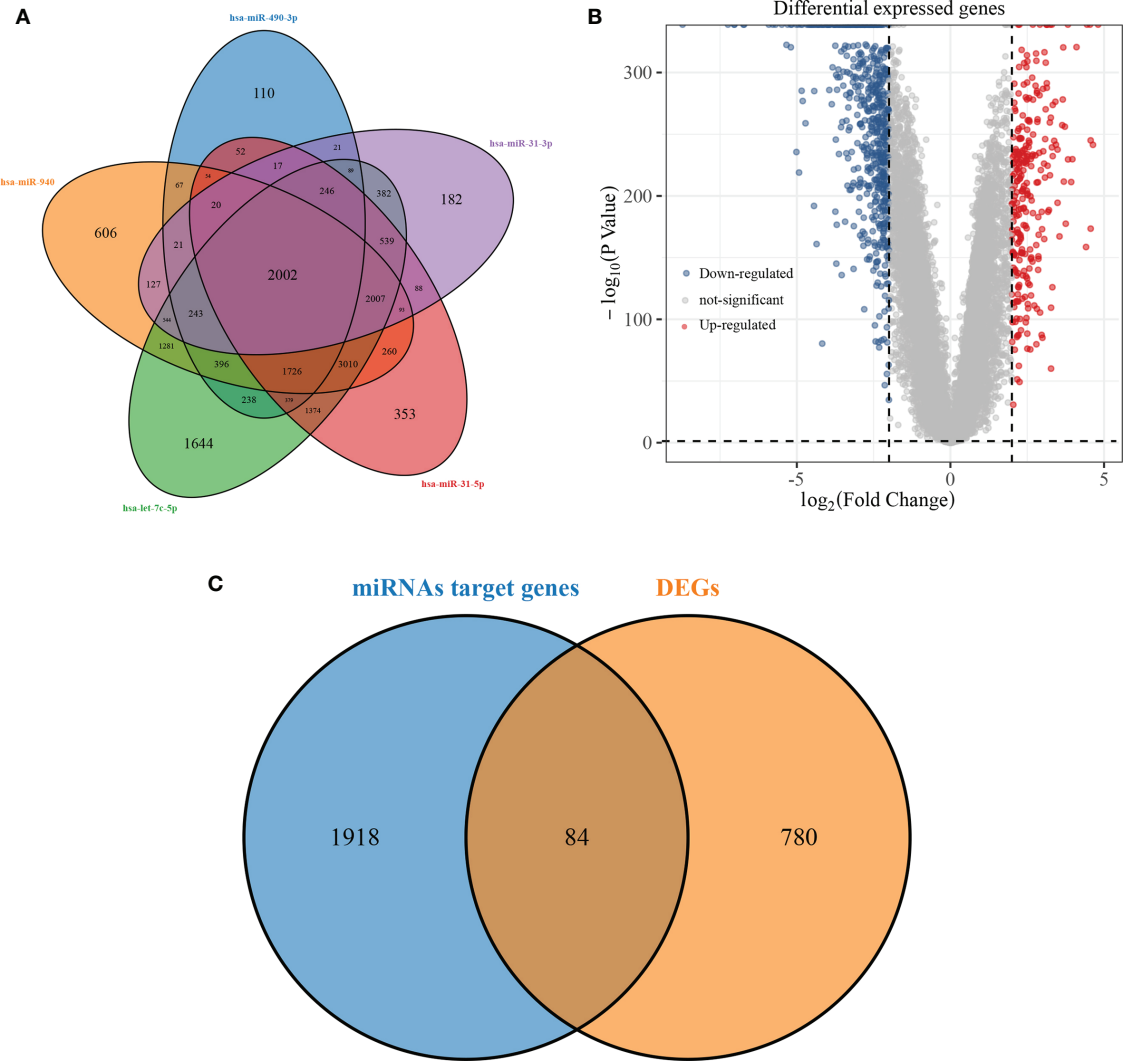
Screening of miRNAs target genes and differentially expressed genes. (**A**: overlapping Venn diagram of predicted target genes for 5 miRNAs; **B**: volcano diagram of differentially expressed genes in LUAD; **C**: overlapping Venn diagram of predicted target genes and differentially expressed genes for miRNAs).

The miRTarBase, TargetScan, and RNA22 databases were used to predict the target genes of hsa-miR-490-3p, hsa-miR-940, hsa-miR-31-3p, hsa-miR-31-5p, and hsa-let-7c-5p, and then the target genes of the five miRNAs were screened, and a total of 2002 intersecting target genes was obtained (**Figure 3A**). Differential analysis of RNA-seq was performed on 513 LUAD tumour samples and 59 normal samples in the TCGA database, and the 256 up-regulated genes and 608 down-regulated genes obtained from the screening were presented using volcano maps (**Figure 3B**). A total of 84 key genes were obtained by overlapping screening of intersecting target genes and differentially expressed genes (**Figure 3C**).

### LASSO Regression Analysis

**Figure 4:**
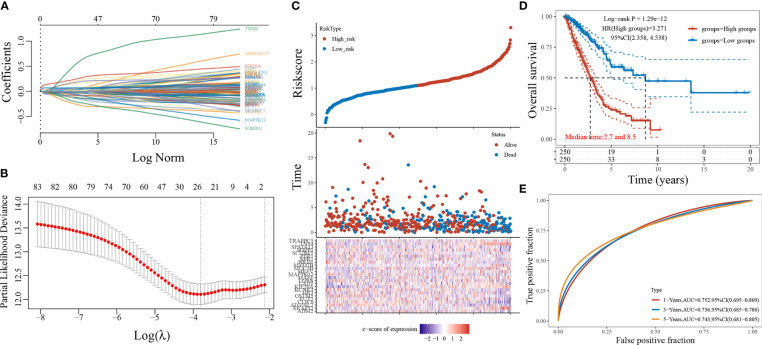
LASSO regression model screening 84 genes. (**A**: different characteristics of genes and their corresponding coefficients; **B**: cross-validation to build the model; **C**: risk score with survival time and survival status cases; **D**: KM curve of this prediction model; **E**: ROC curve plotted under the prediction model).

A 24-gene prediction model was obtained by screening 84 genes using LASSO regression analysis (**Figures 4A, B**), and LUAD patients were divided into a high-risk score group and a low-risk score group based on the median value of the risk score (**Figure 4C**). The LASSO regression survival curves (**Figure 4D**) showed that high-risk patients were worse than low-risk patients. The subjects’ working curves (**Figure 4E**) showed that the AUC areas of the 1-, 3-, and 5-year survival time curves were 0.752, 0.736, and 0.743, respectively, indicating that the model has a strong predictive accuracy.

### Kaplan-Meier Method to Validate 24 Genes

**Figure 5:**
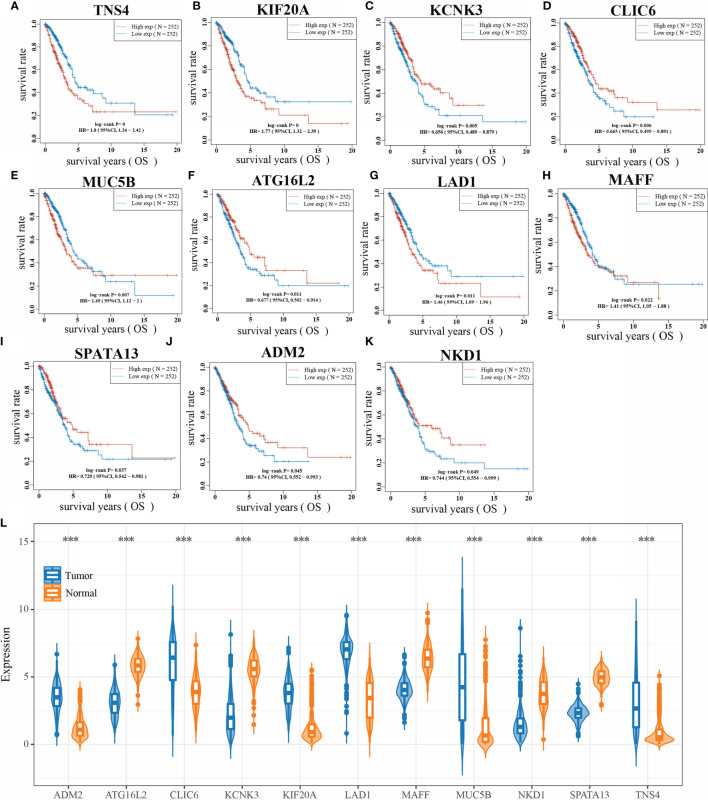
Kaplan-Meier method to validate 23 genes. (**A**: KM curve of TNS4; **B**: KM curve of KIF20A; **C**: KM curve of KCNK3; **D**: KM curve of CLIC6; **E**: KM curve of MUC5B; **F**: KM curve of ATG16L2; **G**: KM curve of LAD1; **H**: KM curve of MAFF; **I**: KM curve of SPATA13; **J**: KM curve of ADM2 curve; **K**: KM curve of NKD1; **L**: expression of 11 genes in LUAD; ***P < 0.0001).

The Kaplan-Meier method was used to validate the overall survival time of 24 key genes, and the hazard coefficients HR of the 95% CI of the 24 genes were obtained by log rank test and univariate Cox proportional hazards regression. Results are presented in **Table 1**, which shows that a total of 11 genes were significantly associated with overall survival in LUAD. Their KM curves are shown in **Figures 5A–K**. The expression of these 11 genes in LUAD is shown in **Figure 5L**. Compared with normal tissues, the expression of ADM2, CLIC6, KIF20A, LAD1, MUC5B, and TNS4 was up-regulated, and the expression of ATG16L2, KCNK3, MAFF, NKD1, and SPATA13 was down-regulated in lung cancer tissues.

**Table 1:** Kaplan-Meier analysis of 24 key genes.

Genes	p.value	HR	95%CI
TNS4	0.0001	1.7963	1.3358-2.416
KIF20A	0.0001	1.7742	1.3189-2.3868
KCNK3	0.0048	0.6560	0.4895-0.8791
CLIC6	0.0064	0.6645	0.4954-0.8914
MUC5B	0.0072	1.4929	1.1146-1.9995
ATG16L2	0.0107	0.6770	0.5018-0.9135
LAD1	0.0109	1.4617	1.0913-1.9577
MAFF	0.0220	1.4058	1.0504-1.8814
SPATA13	0.0368	0.7293	0.5422-0.9809
ADM2	0.0450	0.7405	0.5520-0.9933
NKD1	0.0494	0.7440	0.5540-0.9992
HK3	0.0581	0.7541	0.5632-1.0098
MAP3K12	0.0855	0.7716	0.5742-1.0369
SCUBE1	0.0984	0.7801	0.5821-1.0471
GSTM5	0.1509	0.8076	0.6033-1.0810
PER1	0.2949	1.1680	0.8735-1.5617
MYO7B	0.3126	0.8601	0.6421-1.1523
AKAP12	0.3911	1.1356	0.8492-1.5187
MEFV	0.4173	0.8849	0.6585-1.1891
TRAPPC5	0.5064	1.1039	0.8247-1.4774
SGPP2	0.5445	1.0941	0.8180-1.4635
LGSN	0.6474	1.0701	0.8003-1.4309
NPR1	0.6899	1.0608	0.7937-1.4179
FUT2	0.7921	1.0399	0.7774-1.3910

### Construction of a Risk-Prediction Model Based on 11 Genes

**Figure 6:**
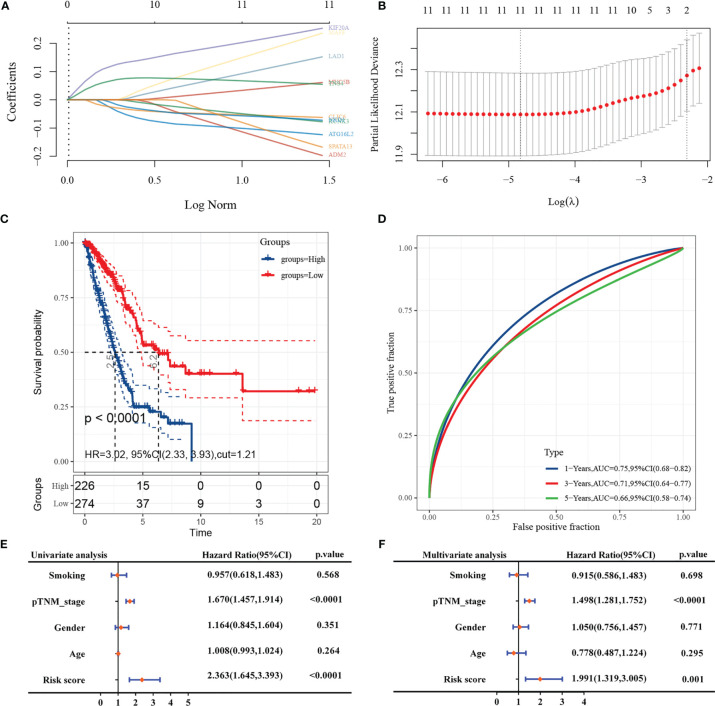
LASSO construction prediction model and COX analysis. (**A**: coefficients of selected characteristics shown by lambda parameters; **B**: partial likelihood deviation versus log(λ); **C**: KM curves for risk grouping; **D**: ROC curves for this risk prediction model; **E**: forest plot for univariate COX regression; **F**: forest plot for multifactor COX regression).

A prediction model based on LASSO regression analysis was constructed for the 11-gene signature (**Figures 6A, B**), and its predicted risk score consisted mainly of the following:


Riskscore=(−0.1707)×ADM2+(−0.0594)×CLIC6+(0.2367)×KIF20A+(0.133)×LAD1+(0.0523)×MUC5B+(0.0594)×TNS4+(−0.1157)×ATG16L2+(−0.0687)×KCNK3+(0.208)×MAFF+(−0.0667)×NKD1+(−0.1406)×SPATA13

The sample was divided into high-risk and low-risk groups according to the risk score ranking, using the best cut-off risk score as the threshold, and the Kaplan-Meier analysis showed that the prognosis of patients in the high-risk group was significantly worse than that of low-risk patients (**Figure 6C**). The subject working characteristic curves indicated that the prediction model had high predictive accuracy for patients’ 1- and 3-year survival prognosis (1-Year AUC: 0.749, 3 Years AUC: 0.708) (**Figure 6D**).

Further GO analysis of the 11 genes showed that the gene signatures were mainly associated with voltage-gated channel activity、S100 protein binding、potassium ion leak channel activity (**Table 2**).

**Table 2:** Significant biological processes in which the 11 gene signatures were mainly involved.

ID	Description	P-value	geneID	Count
GO:0005244	Voltage-gated ion channel activity	5.23E-03	KCNK3/CLIC6	2
GO:0022832	Voltage-gated channel activity	5.23E-03	KCNK3/CLIC6	2
GO:0044548	S100 protein binding	8.45E-03	KCNK3	1
GO:0022841	Potassium ion leak channel activity	9.01E-03	KCNK3	1
GO:0050780	Dopamine receptor binding	9.01E-03	CLIC6	1
GO:0030676	Rac guanyl-nucleotide exchange factor activity	9.57E-03	SPATA13	1
GO:0022840	Leak channel activity	1.07E-02	KCNK3	1
GO:0022842	Narrow pore channel activity	1.07E-02	KCNK3	1
GO:0022839	Ion gated channel activity	1.45E-02	KCNK3/CLIC6	2
GO:0022836	Gated channel activity	1.52E-02	KCNK3/CLIC6	2
GO:0005216	Ion channel activity	2.19E-02	KCNK3/CLIC6	2
GO:0022838	Substrate-specific channel activity	2.31E-02	KCNK3/CLIC6	2
GO:0015267	Channel activity	2.60E-02	KCNK3/CLIC6	2
GO:0022803	Passive transmembrane transporter activity	2.61E-02	KCNK3/CLIC6	2

In univariate Cox regression analysis, pathological TNM stage and risk score was associated with poorer prognosis of patients (**Figure 6E**). In multivariate Cox regression, pathological TNM stage and risk score were identified as independent prognostic predictors (**Figure 6F**).

### Validating the Predictive Value of Risk Model in Independent Cohort

**Figure 7:**
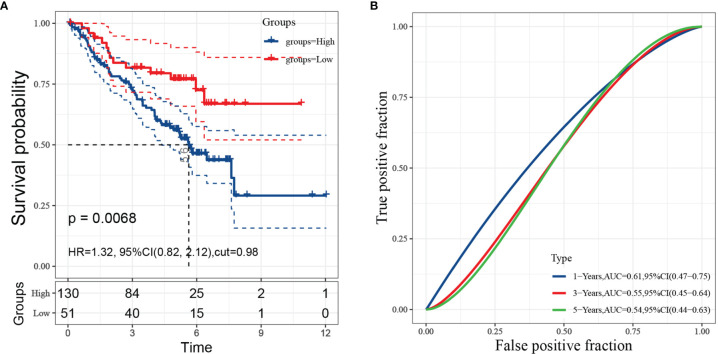
Evaluation of risk model performance in independent datasets. (**A**: Kaplan-Meier curves and univariate Cox regression of overall survival in GSE50081; **B**: ROC curve analyses in GSE50081).

To further verify the predictive value of risk model, GSE50081 from GEO were employed as a validation cohort. Cox regression analysis and the Kaplan-Meier curve showed that high-risk patients were worse than low-risk patients, which agreed with the results found in the TCGA-LUAD cohort (**Figure 7A**). The AUCs of FRRS at 1, 3 and 5 years were 0.61, 0.55, and 0.54, respectively (**Figure 7B**).

### Clinicopathological Characteristics of Different Risk Score Subgroups

**Figure 8:**
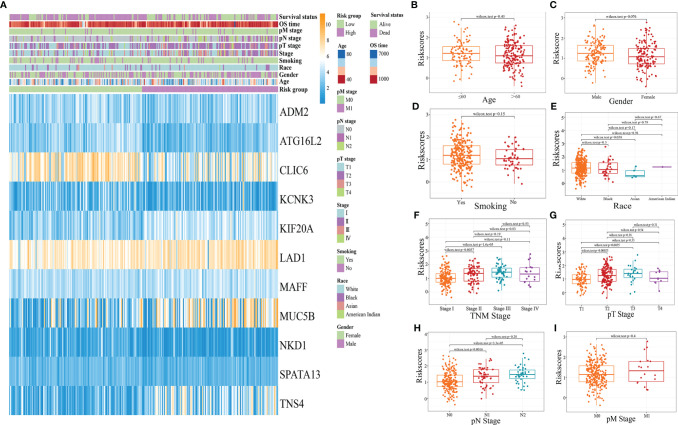
Clinicopathological characteristics of different risk score subgroups. (**A**: heat map of clinicopathological characteristics of different risk score subgroups; **B**: the relationship between age and risk score; **C**: the relationship between sex and risk score; **D**: the relationship between smoking history and Riskscore; **E**: the relationship between race and Riskscore; **F**: the relationship between pathological TNM stage and Riskscore; **G**: the relationship between pathological T stage and Riskscore; **H**;. Relationship between pathological N-stage and Riskscore; **I**: Relationship between pathological M-stage and Riskscore).

The distribution of clinicopathological characteristics of different risk score subgroups (high risk and low risk) was presented as a heat map (**Figure 8A**); the relationship between different age, gender, smoking history, race, pathological TNM stage, pathological T stage, pathological N stage, pathological M stage, and risk score was observed, and the results showed (**Figures 8B–I**) that Stage I patients’ risk score was significantly different from that of Stage II and Stage III patients; the difference between risk score of Stage T1 and T2 and T3 patients was statistically significant. The risk score of Stage N0 patients was significantly different from that of Stage N1 and N2 patients.

## Discussion

miRNAs are not independent regulators, and their action is achieved through binding to target genes. Although the vast majority of genes in the human genome are regulated by the expression of miRNAs, not all of them play a decisive role in tumour development. Therefore, although certain miRNAs are differentially expressed in tumours, it does not mean that this has an impact on tumorigenesis and progression. In this study, we first obtained a total of 123 up-regulated miRNAs and 22 down-regulated miRNAs, 256 up-regulated genes and 608 down-regulated genes by screening differentially expressed miRNAs and genes in LUAD. We then further subjected these miRNAs to LASSO regression analysis and validated them with KM to obtain a total of five prognosis-related LUAD patients. These five miRNAs have been shown to be differentially expressed in LUAD by bioinformatics analysis in previous studies (22). Subsequently, we predicted the target genes of these five miRNAs and found that there were 2002 target genes in which the five miRNAs acted together, and a total of 84 genes were obtained by screening them with overlapping differentially expressed genes in LUAD. The vast majority of studies using training groups to develop and construct molecular markers rely on the selection of overlapping genes in multiple databases, which may lead to the recurrence of certain genes in new signatures, a phenomenon that may lead to similarity or convergence of results and may hinder the efficiency and predictive power of prediction models (23). To improve our accuracy, we performed downscaling and validation of 84 genes to finally construct a risk-prediction model with 11 gene signatures consisting of 11 genes significantly associated with LUAD prognosis. This 11-gene signature risk-prediction model classified patients into high-risk and low-risk groups, and there was a significant difference in the overall survival prognosis between the high- and low-risk groups. This model could be a valid and promising prognostic biomarker for lung adenocarcinoma patients as an independent prognostic predictor. Previously studies also constructed a series of models to predicting prognosis in LUAD *via* different bioinformatic analysis. Zhou C et al. (24) constructed prognosis predicting risk model based on platelet-related gene expression. Besides this, Ye GC et al. (25) identified key microRNAs and hub genes associated with poor prognosis in lung adenocarcinoma *via* miRNA-mRNA network, they indicated that PECAM1, in particular, may be a novel biomarker of survival that provided a novel diagnostic biomarkers and therapeutic targets for the treatment of LUAD. Except for miRNA or mRNA, lncRNA also plays an important role in the pathogenesis of cancer and has significant clinical value in prognosis and diagnosis (26). Li R et al. (27) applied an integrated ceRNA network analysis to identify a lncRNA-based signature for predicting the prognosis of LUAD patients. The established molecular signature with seven lncRNAs, derived from the ceRNA network, was demonstrated to be a robust and independent factor for the survival prediction of LUAD patients.

The 11 gene signatures are composed of ADM2, CLIC6, KIF20A, LAD1, MUC5B, TNS4, ATG16L2, KCNK3, MAFF, NKD1, and SPATA13. adrenomedullin-2 (ADM2) is a hypoxia-inducible endothelial peptide that stabilizes pulmonary microvascular.CLIC6 is a member of the intracellular chloride channel, one of the dopamine receptor-mediated signalling pathways, and is differentially expressed in breast cancer. Recent studies have reported evidence that CLIC6 expression is significantly associated with lung adenocarcinoma prognosis (28, 29). KIF20A (30), LAD1 (31), MUC5B (32), TNS4 (33), ATG16L2 (34), and NKD1 (35) have been shown to serve as biological markers for prognosis prediction in lung cancer. KCNK3 (36) has been reported to be involved in pulmonary hypertension, which may contribute to poor prognosis in lung cancer patients. SPATA13 is a discrete region in the adult brain enriched in a guanylate exchange factor (37), and deletion of SPATA13 has been shown to reduce and shrink the number and size of intestinal tumours in Apc (Min/+) mice (38, 39), but its study in lung cancer is rare. In addition to this, we investigated the risk scores in different LUAD clinicopathological features. The results suggest that the risk score of Stage I in patients with different TNM stages is significantly different from that of Stage II, III, and IV, and the same situation applies to different T and N stages. This result also demonstrates that this prediction model may serve as a biological marker for the early diagnosis of LUAD.

In conclusion, a valid and accurate prognostic model for LUAD based on 11 gene signatures was constructed in this study. The prediction model based on these 11 gene signatures has good predictive properties, and it can effectively distinguish between high-risk and low-risk patients based on risk scores. There are significant differences in its risk scores among patients with different TNM stages, making it an early diagnostic marker and prognostic predictor for LUAD patients and reducing the excessive cost of molecular diagnosis. However, there are some limitations to this study. First, since our study relied mainly on bioinformatics analysis, these results subsequently require a series of biological experiments to assist validation. The potential biological mechanisms and pathways associated with these 11 genes still need further investigation.

## Data Availability Statement

The original contributions presented in the study are included in the article/supplementary material. Further inquiries can be directed to the corresponding author.

## Author Contributions

All authors listed have made a substantial, direct, and intellectual contribution to the work and approved it for publication.

## Conflict of Interest

The authors declare that the research was conducted in the absence of any commercial or financial relationships that could be construed as a potential conflict of interest.

## Publisher’s Note

All claims expressed in this article are solely those of the authors and do not necessarily represent those of their affiliated organizations, or those of the publisher, the editors and the reviewers. Any product that may be evaluated in this article, or claim that may be made by its manufacturer, is not guaranteed or endorsed by the publisher.
